# Public Health Responses to COVID-19 Outbreaks on Cruise Ships — Worldwide, February–March 2020

**DOI:** 10.15585/mmwr.mm6912e3

**Published:** 2020-03-27

**Authors:** Leah F. Moriarty, Mateusz M. Plucinski, Barbara J. Marston, Ekaterina V. Kurbatova, Barbara Knust, Erin L. Murray, Nicki Pesik, Dale Rose, David Fitter, Miwako Kobayashi, Mitsuru Toda, Paul T. Canty, Tara Scheuer, Eric S. Halsey, Nicole J. Cohen, Lauren Stockman, Debra A. Wadford, Alexandra M. Medley, Gary Green, Joanna J. Regan, Kara Tardivel, Stefanie White, Clive Brown, Christina Morales, Cynthia Yen, Beth Wittry, Amy Freeland, Sara Naramore, Ryan T. Novak, David Daigle, Michelle Weinberg, Anna Acosta, Carolyn Herzig, Bryan K Kapella, Kathleen R. Jacobson, Katherine Lamba, Atsuyoshi Ishizumi, John Sarisky, Erik Svendsen, Tricia Blocher, Christine Wu, Julia Charles, Riley Wagner, Andrea Stewart, Paul S. Mead, Elizabeth Kurylo, Stefanie Campbell, Rachel Murray, Paul Weidle, Martin Cetron, Cindy R. Friedman, CDC Cruise Ship Response Team, California Department of Public Health COVID-19 Team, Solano County COVID-19 Team, CDC Cruise Ship Response Team, Casey Barton Behravesh, Adam Bjork, William Bower, Catherine Bozio, Zachary Braden, Mary Catherine Bertulfo, Kevin Chatham-Stephens, Victoria Chu, Barbara Cooper, Kathleen Dooling, Christine Dubray, Emily Curren, Margaret A. Honein, Kathryn Ivey, Jefferson Jones, Melissa Kadzik, Nancy Knight, Mariel Marlow, Audrey McColloch, Robert McDonald, Andrew Klevos, Sarah Poser, Robin A. Rinker, Troy Ritter, Luis Rodriguez, Matthew Ryan, Zachary Schneider, Caitlin Shockey, Jill Shugart, Margaret Silver, Paul W. Smith, Farrell Tobolowsky, Aimee Treffiletti, Megan Wallace, Jonathan Yoder, California Department of Public Health COVID-19 Team, Pennan Barry, Ricardo Berumen, Brooke Bregman, Kevin Campos, Shua Chai, Rosie Glenn-Finer, Hugo Guevara, Jill Hacker, Kristina Hsieh, Mary Kate Morris, Ryan Murphy, Jennifer F. Myers, Tasha Padilla, Chao-Yang Pan, Adam Readhead, Estela Saguar, Maria Salas, Robert E. Snyder, Duc Vugia, James Watt, Cindy Wong, Solano County COVID-19 Team, Meileen Acosta, Shai Davis, Beatrix Kapuszinsky, Bela Matyas, Glen Miller, Asundep Ntui, Jayleen Richards

**Affiliations:** ^1^CDC COVID-19 Response Team; ^2^California Department of Public Health; ^3^Solano Public Health, Fairfield, California; ^4^Epidemic Intelligence Service, CDC; ^5^Sutter Medical Group of the Redwoods, Santa Rosa, California.; CDC; CDC; CDC; CDC; CDC; CDC; CDC; CDC; CDC; CDC; CDC; CDC; CDC; CDC; CDC; CDC; CDC; CDC; CDC; CDC; CDC; CDC; CDC; CDC; CDC; CDC; CDC; CDC; CDC; CDC; CDC; CDC; CDC; CDC; CDC.; California Department of Public Health; California Department of Public Health; California Department of Public Health; California Department of Public Health; California Department of Public Health; California Department of Public Health; California Department of Public Health; California Department of Public Health; California Department of Public Health; California Department of Public Health; California Department of Public Health; California Department of Public Health; California Department of Public Health; California Department of Public Health; California Department of Public Health; California Department of Public Health; California Department of Public Health; California Department of Public Health; California Department of Public Health; California Department of Public Health; California Department of Public Health.; Solano County Department of Public Health; Solano County Department of Public Health; Solano County Department of Public Health; Solano County Department of Public Health; Solano County Department of Public Health; Solano County Department of Public Health; Solano County Department of Public Health.

An estimated 30 million passengers are transported on 272 cruise ships worldwide each year* (*1*). Cruise ships bring diverse populations into proximity for many days, facilitating transmission of respiratory illness (*2*). SARS-CoV-2, the virus that causes coronavirus disease (COVID-19) was first identified in Wuhan, China, in December 2019 and has since spread worldwide to at least 187 countries and territories. Widespread COVID-19 transmission on cruise ships has been reported as well (*3*). Passengers on certain cruise ship voyages might be aged ≥65 years, which places them at greater risk for severe consequences of SARS-CoV-2 infection (*4*). During February–March 2020, COVID-19 outbreaks associated with three cruise ship voyages have caused more than 800 laboratory-confirmed cases among passengers and crew, including 10 deaths. Transmission occurred across multiple voyages of several ships. This report describes public health responses to COVID-19 outbreaks on these ships. COVID-19 on cruise ships poses a risk for rapid spread of disease, causing outbreaks in a vulnerable population, and aggressive efforts are required to contain spread. All persons should defer all cruise travel worldwide during the COVID-19 pandemic.

During February 7–23, 2020, the largest cluster of COVID-19 cases outside mainland China occurred on the Diamond Princess cruise ship, which was quarantined in the port of Yokohama, Japan, on February 3 (*3*). On March 6, cases of COVID-19 were identified in persons on the Grand Princess cruise ship off the coast of California; that ship was subsequently quarantined. By March 17, confirmed cases of COVID-19 had been associated with at least 25 additional cruise ship voyages. On February 21, CDC recommended avoiding travel on cruise ships in Southeast Asia; on March 8, this recommendation was broadened to include deferring all cruise ship travel worldwide for those with underlying health conditions and for persons aged ≥65 years. On March 13, the Cruise Lines International Association announced a 30-day voluntary suspension of cruise operations in the United States (*5*). CDC issued a level 3 travel warning on March 17, recommending that all cruise travel be deferred worldwide.^†^

## Diamond Princess

On January 20, 2020, the Diamond Princess cruise ship departed Yokohama, Japan, carrying approximately 3,700 passengers and crew (Table). On January 25, a symptomatic passenger departed the ship in Hong Kong, where he was evaluated; testing confirmed SARS-CoV-2 infection. On February 3, the ship returned to Japan, after making six stops in three countries. Japanese authorities were notified of the COVID-19 diagnosis in the passenger who disembarked in Hong Kong, and the ship was quarantined. Information about social distancing and monitoring of symptoms was communicated to passengers. On February 5, passengers were quarantined in their cabins; crew continued to work and, therefore, could not be isolated in their cabins (*6*). Initially, travelers with fever or respiratory symptoms and their close contacts were tested for SARS-CoV-2 by reverse transcription–polymerase chain reaction (RT-PCR). All those with positive test results were disembarked and hospitalized. Testing was later expanded to support a phased disembarkation of passengers, prioritizing testing of older persons, those with underlying medical conditions, and those in internal cabins with no access to the outdoors. During February 16–23, nearly 1,000 persons were repatriated by air to their home countries, including 329 persons who returned to the United States and entered quarantine or isolation.^§^^,^^¶^

**TABLE:** Demographic characteristics of passengers and crew members on board two cruise ships with COVID-19 outbreaks January 20–March 8, 2020

Characteristic	Diamond Princess (total 3,711 persons)		Grand Princess, voyage B (total 3,571 persons)	
	Crew	Passengers	Crew	Passengers
**Total no.**	**1,045**	**2,666**	**1,111**	**2,460**
**Age median (interquartile range), yrs**	36 (29–43)	69 (62–73)	36 (30–43)	68 (61–74)
**Total nations represented**	48	36	44	24
**Country of residence of passengers, no. (%)**				
Japan	N/A	1,281 (48)	N/A	3 (1)
United States	N/A	416 (16)	N/A	2,008 (82)
Hong Kong	N/A	260 (10)	N/A	0 (0)
Canada	N/A	251 (9)	N/A	231 (9)
Australia	N/A	223 (8)	N/A	1 (0)
United Kingdom	N/A	57 (2)	N/A	113 (4)
Other countries or unknown	N/A	178 (7)	N/A	104 (4)
**Country of residence of crew members, no. (%)**				
Philippines	531 (51)	N/A	529 (48)	N/A
India	132 (13)	N/A	131 (12)	N/A
Indonesia	78 (7)	N/A	57 (5)	N/A
Other countries or unknown	304 (29)	N/A	394 (35)	N/A
**Sex, no. (%)**				
Male	843 (81)	1,189 (45)	928 (84)	1,120 (46)
Female	202 (19)	1,477 (55)	183 (16)	1,340 (54)
**No. of persons per cabin, mean (range)**	1.73 (1–3)	1.98 (1–4)	1.75 (1–4)	1.95 (1–4)

**Abbreviation:** N/A = not applicable.

The remaining passengers who had negative SARS-CoV-2 RT-PCR test results,** no respiratory symptoms, and no close contact with a person with a confirmed case of COVID-19 completed a 14-day ship-based quarantine before disembarkation. Those passengers who had close contact with a person with a confirmed case completed land-based quarantine, with duration determined by date of last contact. After disembarkation of all passengers, crew members either completed a 14-day ship-based quarantine, were repatriated to and managed in their home country, or completed a 14-day land-based quarantine in Japan.

Overall, 111 (25.9%) of 428 U.S. citizens and legal residents did not join repatriation flights either because they had been hospitalized in Japan or for other reasons. To mitigate SARS-CoV-2 importation into the United States, CDC used temporary “Do Not Board” restrictions (*7*) to prevent commercial airline travel to the United States,^††^ and the U.S. Departments of State and Homeland Security restricted travel to the United States for non-U.S. travelers.

**FIGURE 1:**
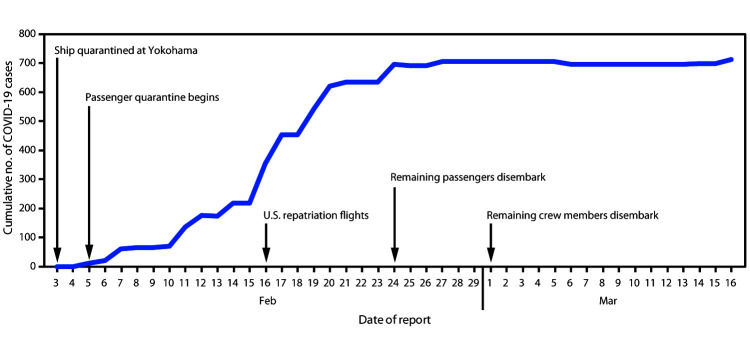
Cumulative number of confirmed coronavirus disease 2019 (COVID-19) cases* by date of detection — Diamond Princess cruise ship, Yokohama, Japan, February 3–March 16, 2020 **Source:** World Health Organization (WHO) coronavirus disease (COVID-2019) situation reports. https://www.who.int/emergencies/diseases/novel-coronavirus-2019/situation-reports/. * Decline in cumulative number of cases on February 13 and February 25 due to correction by WHO for cases that had been counted twice.

Among 3,711 Diamond Princess passengers and crew, 712 (19.2%) had positive test results for SARS-CoV-2 (Figure 1). Of these, 331 (46.5%) were asymptomatic at the time of testing. Among 381 symptomatic patients, 37 (9.7%) required intensive care, and nine (1.3%) died (*8*). Infections also occurred among three Japanese responders, including one nurse, one quarantine officer, and one administrative officer (*9*). As of March 13, among 428 U.S. passengers and crew, 107 (25.0%) had positive test results for COVID-19; 11 U.S. passengers remain hospitalized in Japan (median age = 75 years), including seven in serious condition (median age = 76 years).

## Grand Princess

During February 11–21, 2020, the Grand Princess cruise ship sailed roundtrip from San Francisco, California, making four stops in Mexico (voyage A). Most of the 1,111 crew and 68 passengers from voyage A remained on board for a second voyage that departed San Francisco on February 21 (voyage B), with a planned return on March 7 (Table). On March 4, a clinician in California reported two patients with COVID-19 symptoms who had traveled on voyage A, one of whom had positive test results for SARS-CoV-2. CDC notified the cruise line, which began cancelling group activities on voyage B. More than 20 additional cases of COVID-19 among persons who did not travel on voyage B have been identified from Grand Princess voyage A, the majority in California. One death has been reported. On March 5, a response team was transported by helicopter to the ship to collect specimens from 45 passengers and crew with respiratory symptoms for SARS-CoV-2 testing; 21 (46.7%), including two passengers and 19 crew, had positive test results. Passengers and symptomatic crew members were asked to self-quarantine in their cabins, and room service replaced public dining until disembarkation. Following docking in Oakland, California, on March 8, passengers and crew were transferred to land-based sites for a 14-day quarantine period or isolation. Persons requiring medical attention for other conditions or for symptoms consistent with COVID-19 were evaluated, tested for SARS-CoV-2 infection, and hospitalized if indicated. During land-based quarantine in the United States, all persons were offered SARS-CoV-2 testing. As of March 21, of 469 persons with available test results, 78 (16.6%) had positive test results for SARS-CoV-2. Repatriation flights for foreign nationals were organized by several governments in coordination with U.S. federal and California state government agencies. Following disinfection of the vessel according to guidance from CDC’s Vessel Sanitation Program, remaining foreign nationals will complete quarantine on board. The quarantine will be managed by the cruise company, with technical assistance provided by public health experts.

On February 21, five crew members from voyage A transferred to three other ships with a combined 13,317 passengers on board. No-sail orders^§§^ were issued by CDC for these ships until medical logs were reviewed and the crew members tested negative for SARS-CoV-2.

## Additional Ships

**FIGURE 2:**
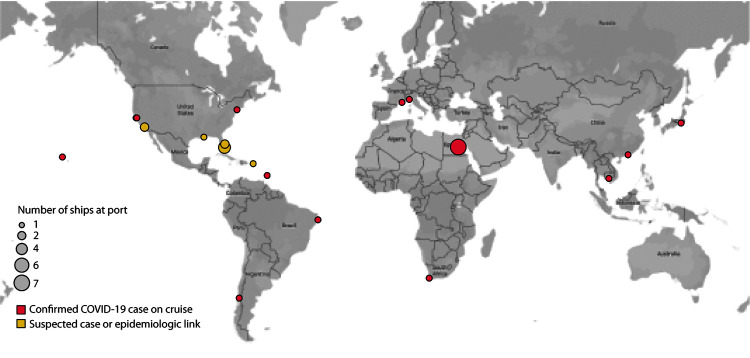
Cruise ships with coronavirus disease 2019 (COVID-19) cases requiring public health responses — worldwide, January–March 2020

The Diamond Princess and Grand Princess had more than 800 total COVID-19 cases, including 10 deaths. During February 3–March 13, in the United States, approximately 200 cases of COVID-19 were confirmed among returned cruise travelers from multiple ship voyages, including the Diamond Princess and Grand Princess, accounting for approximately 17% of total reported U.S. cases at the time (*10*). Cases linked with cruise travel have been reported to CDC in at least 15 states. Since February, multiple international cruises have been implicated in reports of COVID-19 cases, including at least 60 cases in the United States from Nile River cruises in Egypt (Figure 2). Secondary community-acquired cases linked to returned passengers on cruises have also been reported (CDC, unpublished data, 2020).

## Discussion

Public health responses to COVID-19 outbreaks on cruise ships were aimed at limiting transmission among passengers and crew, preventing exportation of COVID-19 to other communities, and assuring the safety of travelers and responders. These responses required the coordination of stakeholders across multiple sectors, including U.S. Government departments and agencies, foreign ministries of health, foreign embassies, state and local public health departments, hospitals, laboratories, and cruise ship companies. At the time of the Diamond Princess outbreak, it became apparent that passengers disembarking from cruise ships could be a source of community transmission. Therefore, aggressive efforts to contain transmission on board and prevent further transmission upon disembarkation and repatriation were instituted. These efforts included travel restrictions applied to persons, movement restrictions applied to ships, infection prevention and control measures, (e.g., use of personal protective equipment for medical and cleaning staff), disinfection of the cabins of persons with suspected COVID-19, provision of communication materials, notification of state health departments, and investigation of contacts of cases identified among U.S. returned travelers.

Cruise ships are often settings for outbreaks of infectious diseases because of their closed environment, contact between travelers from many countries, and crew transfers between ships. On the Diamond Princess, transmission largely occurred among passengers before quarantine was implemented, whereas crew infections peaked after quarantine (*6*). On the Grand Princess, crew members were likely infected on voyage A and then transmitted SARS-CoV-2 to passengers on voyage B. The results of testing of passengers and crew on board the Diamond Princess demonstrated a high proportion (46.5%) of asymptomatic infections at the time of testing. Available statistical models of the Diamond Princess outbreak suggest that 17.9% of infected persons never developed symptoms (*9*). A high proportion of asymptomatic infections could partially explain the high attack rate among cruise ship passengers and crew. SARS-CoV-2 RNA was identified on a variety of surfaces in cabins of both symptomatic and asymptomatic infected passengers up to 17 days after cabins were vacated on the Diamond Princess but before disinfection procedures had been conducted (Takuya Yamagishi, National Institute of Infectious Diseases, personal communication, 2020). Although these data cannot be used to determine whether transmission occurred from contaminated surfaces, further study of fomite transmission of SARS-CoV-2 aboard cruise ships is warranted.

During the initial stages of the COVID-19 pandemic, the Diamond Princess was the setting of the largest outbreak outside mainland China. Many other cruise ships have since been implicated in SARS-CoV-2 transmission. Factors that facilitate spread on cruise ships might include mingling of travelers from multiple geographic regions and the closed nature of a cruise ship environment. This is particularly concerning for older passengers, who are at increased risk for serious complications of COVID-19 (*4*). The Grand Princess was an example of perpetuation of transmission from crew members across multiple consecutive voyages and the potential introduction of the virus to passengers and crew on other ships. Public health responses to cruise ship outbreaks require extensive resources. Temporary suspension of cruise ship travel during the current phase of the COVID-19 pandemic has been partially implemented by cruise lines through voluntary suspensions of operations, and by CDC through its unprecedented use of travel notices and warnings for conveyances to limit disease transmission (*5*).

**Summary**

**What is already known about this topic?**

Cruise ships are often settings for outbreaks of infectious diseases because of their closed environment and contact between travelers from many countries.

**What is added by this report?**

More than 800 cases of laboratory-confirmed COVID-19 cases occurred during outbreaks on three cruise ship voyages, and cases linked to several additional cruises have been reported across the United States. Transmission occurred across multiple voyages from ship to ship by crew members; both crew members and passengers were affected; 10 deaths associated with cruise ships have been reported to date.

**What are the implications for public health practice?**

Outbreaks of COVID-19 on cruise ships pose a risk for rapid spread of disease beyond the voyage. Aggressive efforts are required to contain spread. All persons should defer all cruise travel worldwide during the COVID-19 pandemic.

## Footnotes

* Not including river cruises.

† Warning level 3: avoid non-essential travel due to widespread ongoing transmission: https://wwwnc.cdc.gov/travel/notices/warning/novel-coronavirus-china.

§ Quarantine was used for persons who were exposed; isolation was used for persons who had positive test results for SARS-CoV-2.

¶ Movement for one person with resolved COVID-19 was not restricted.

** Based on Japanese testing procedures, which at the time included taking one oropharyngeal swab.

†† Travel restrictions were lifted when persons had either completed a 14-day monitoring period without symptoms or had met clinical criteria for release from isolation. https://japan2.usembassy.gov/pdfs/alert-20200227-diamond-princess.pdf.

§§ CDC has the authority to institute a no-sail order to prevent ships from sailing when it is reasonably believed that continuing normal operations might subject newly arriving passengers to disease.
